# Enzymatic blood group conversion of human kidneys during *ex vivo* normothermic machine perfusion

**DOI:** 10.1093/bjs/znac293

**Published:** 2022-08-30

**Authors:** Serena MacMillan, Sarah A Hosgood, Michael L Nicholson

**Affiliations:** Department of Surgery, University of Cambridge, Cambridge, UK; Department of Surgery, University of Cambridge, Cambridge, UK; Department of Surgery, University of Cambridge, Cambridge, UK

## Abstract

A major restriction to transplantation is the requirement for ABO blood group compatibility between donor and recipient. In this study, an α-galactosidase enzyme from *Bacteroides fragilis* was used successfully to remove type B blood group antigens enzymatically from human kidneys using *ex vivo* normothermic machine perfusion. This provides the first step for a strategy to overcome the ABO barrier in kidney transplantation.

## Introduction

Kidney transplantation remains the best therapeutic option for patients with end-stage kidney disease, and yet a major restriction to transplantation is the requirement for ABO blood group compatibility between donor and recipient. This requirement prevents the recipient’s pre-existing circulating antibodies from initiating antibody-mediated damage to a graft expressing incompatible antigens, which may lead, in extreme cases, to hyperacute rejection^1–3^. This short report details, for the first time, the successful use of an α-galactosidase enzyme to remove type B blood group antigens from human kidneys as a strategy for overcoming the ABO barrier in kidney transplantation.

The blood group antigens that initiate immunological responses after transplantation are found on the cells of the vascular endothelium, among other tissues^4,5^. These antigens consist of a core oligosaccharide precursor chain called the H antigen, and the subsequent addition of a terminal *N*-acetylgalactosamine or galactose sugar produces the immunogenic type A or type B antigen respectively. From early childhood, individuals produce antibodies against non-native blood group antigens, meaning that individuals of blood group A produce antibodies against blood group B, and vice versa. Solid organ transplantation largely involves ABO-compatible (ABOc) transplants. A small percentage of kidney transplants are intentionally ABO-incompatible (ABOi), requiring pretransplant desensitization of the recipient by plasmapheresis (or immunoadsorption) and an intensive immunosuppression regimen^6–8^. Because of these factors, ABOi kidney transplants are almost exclusively restricted to living donors.

Recent research efforts have explored techniques to modify ABO blood group antigens in human tissues^9–12^. One such method involves the enzymatic digestion of the terminal monosaccharides from blood group antigens to remove the immunogenic sugars. In the context of solid organ transplantation, cleavage of the blood group A and B antigens on the vascular endothelium by so-named A- or B-zymes would produce non-immunogenic H antigens, and thus make the organ universally transplantable into any recipient. Success has been shown recently with the use of *ex vivo* normothermic machine perfusion (NMP) to facilitate the administration of A-zymes to convert human lungs from blood group A to O^12^. The first example of *ex vivo* enzymatic blood group conversion of human blood group B kidneys is provided here as proof-of-principle work toward universal donor organs.

## Methods and results

### Blood group B antigen removal *in vitro*

Schematics of the study design and the principle of blood group antigen removal are outlined in *Figs S1* and *S2* respectively. For evaluating blood group B antigen removal *in vitro,* five human kidney cortex biopsies from blood group B-positive kidneys underwent incubation with serially diluted concentrations of an α-galactosidase enzyme (GH110B) from *Bacteroides fragilis* for 1 h at 37°C; further details are available in the *supplementary material* .

**Fig. 1:**
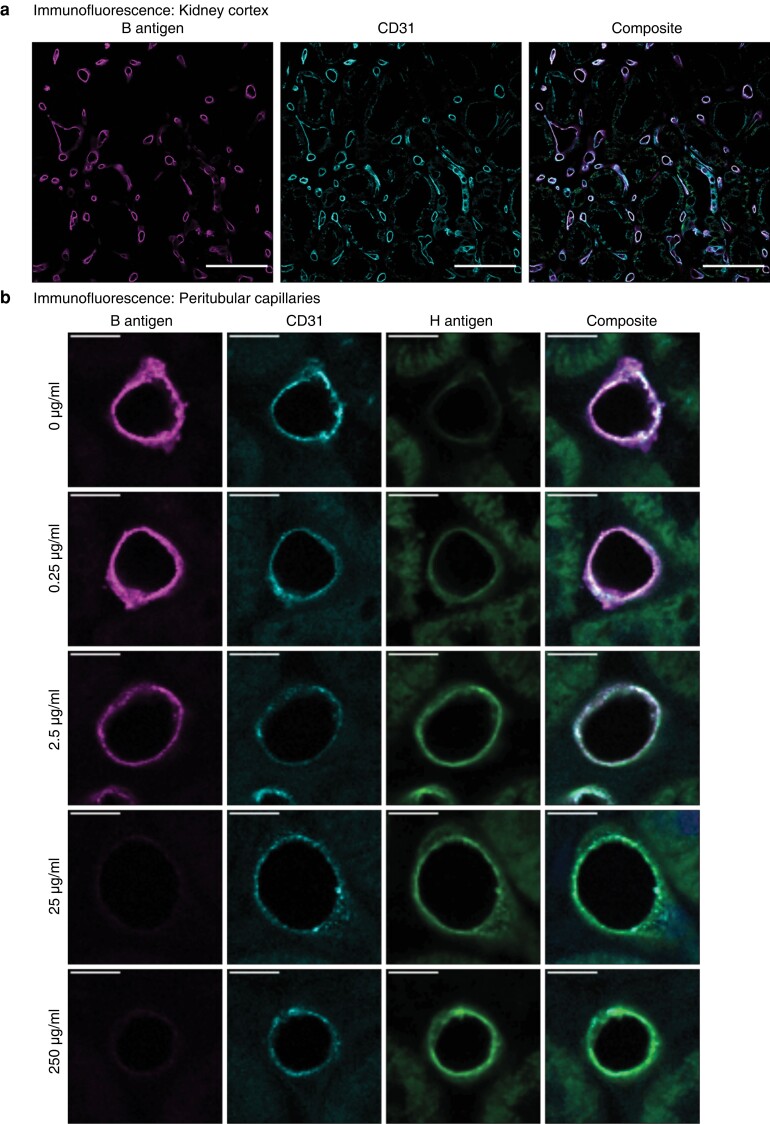

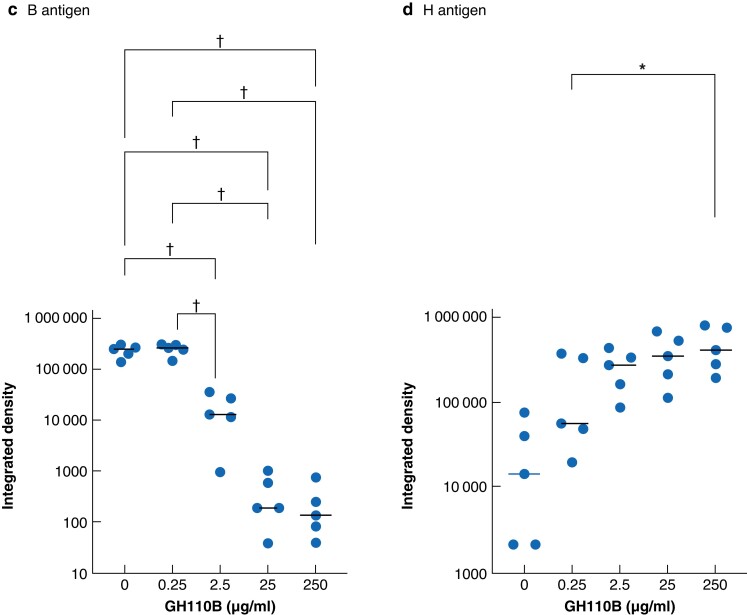
Enzymatic removal of B antigens *in vitro*

Strong anti-B immunofluorescence staining of the peritubular capillaries was observed in the untreated sections (*Fig. 1a*). After incubation with 2.5 μg/ml GH110B, quantification of anti-B staining showed a significant decrease of 93 per cent compared with untreated controls (*P* = 0.005). A maximum of 99 per cent antigen removal was achieved at a concentration of 250 μg/ml (*P* = 0.006), with no significant difference in B antigen loss between sections treated with 2.5, 25 or 250 μg/ml GH110B (*Fig. 1b*,*c* ). H antigen staining with the lectin *Ulex europaeus* increased with the addition of all concentrations of GH110B, although no significant difference was observed between untreated and treated sections (*Fig. 1b*,*d* ). As the lowest concentration of enzyme to show significant antigen loss after 1 h was 2.5 μg/ml, this concentration was chosen for trialling *ex vivo*.

**a** Immunofluorescence staining of a human blood group B cortical kidney biopsy section, stained for B antigens (left-hand panel) and CD31 (middle panel). A composite of overlayed images is shown in the right-hand panel. Scale bar represents 100 μm. **b** Immunofluorescence images showing representative peritubular capillaries stained for blood group B antigens (first column), CD31 (second column), and H antigens (third columnn) after incubation of human kidney cortex ormalin-fixed paraffin-embedded sections with different concentrations of GH110B (0–250μg/ml). A composite of overlayed images is shown in the right-hand panel. Scale bar represents 10 μm. **c**,**d** Quantification of **c** B antigen and **d** H antigen staining (*n* = 5 biological replicates). Bars represent median values and dots represent individual biological replicates. *P < 0.050, †*P* < 0.010 (one-way repeated measure ANOVA with post-hoc Tukey's multiple comparisons test).

### Blood group conversion of whole human kidney during *ex vivo* normothermic perfusion

Three human donor kidneys rejected for transplantation and offered for research (2 donation after brain death, DBD; 1 donation after circulatory death, DCD) were perfused with an acellular perfusate supplemented with 2.5 μg/ml GH110B for 5 h at 37°C using NMP (kidneys 1–3). The pair of one of the treated kidneys was perfused in similar conditions without enzyme addition as a control (kidney 4; DBD). Details of donor characteristics and perfusion parameters are outlined in *Tables S1*, *S2* and *Figs S3*, *S4* .

**Fig. 2:**
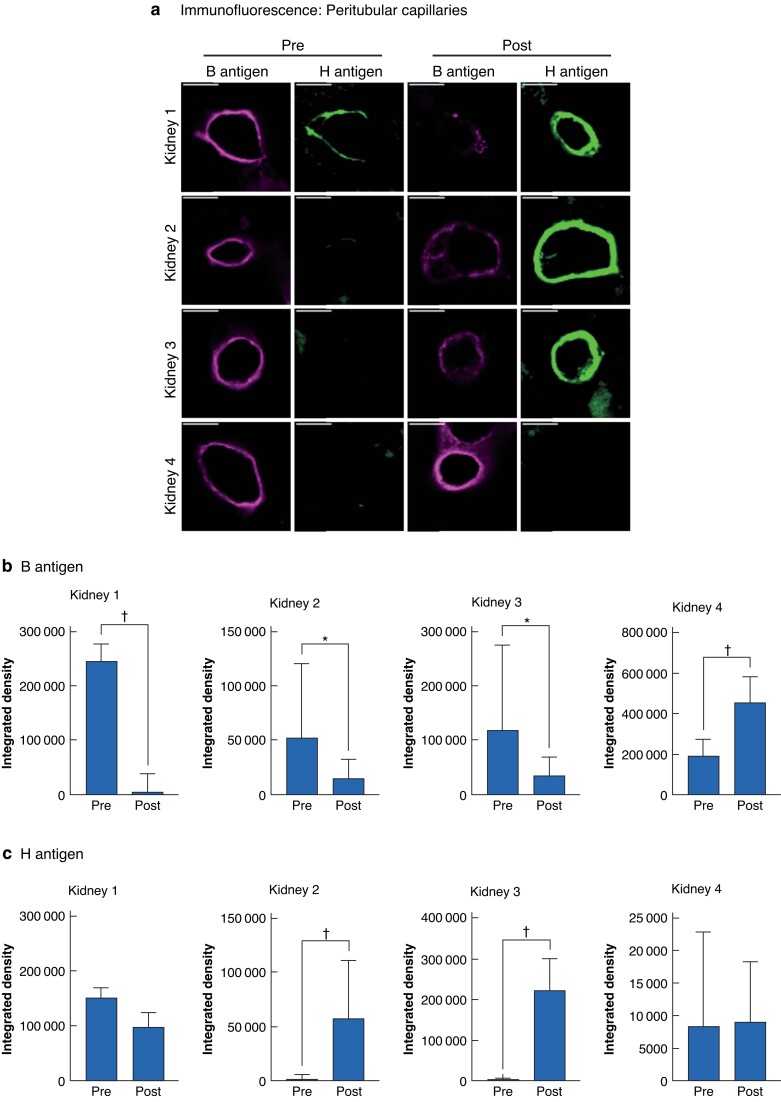
Enzymatic B antigen removal in whole kidneys during *ex vivo* normothermic machine perfusion

Cortical wedge biopsies taken hourly during perfusion were evaluated with immunofluorescence staining of B and H antigens (*Fig. 2a*). Maximal antigen removal was achieved after 5 h of NMP for all kidneys compared with control biopsies taken before addition of enzyme (*Fig. S5*). For kidney 1, 94 per cent of B antigens were removed (*P* = 0.001); 67 per cent of antigens were removed from kidney 2 (*P* = 0.011) and 71 per cent from kidney 3 (*P* = 0.045) after 5 h of perfusion (*Fig. 2b*). Conversely, for the untreated control kidney (kidney 4; biological pair of kidney 3), B antigen staining was more intense after NMP, likely owing to vascular dilatation during perfusion increasing the fluorescence intensity. H antigen emergence followed inversely, increasing by 25.7-fold in kidney 2 (*P* < 0.001) and by 66.0-fold in kidney 3 (*P* < 0.001) after NMP compared with levels in biopsies taken before addition of enzyme (*Fig. 2a*,*c* ). No significant difference was observed in H antigen expression in kidney 1 (*P* = 0.073) or the untreated kidney 4 (*P* = 0.948). Notably, kidney 1 showed high levels of H antigen staining in the biopsy taken before addition of enzyme, reflecting a higher level of native H antigen expression before treatment (*Fig. 2a*).

**a** Immunofluorescence images showing representative peritubular capillary staining of blood group B antigens (first and third columns) and H antigens (second and fourth columns) before addition of enzyme (pre), and after 5 h of normothermic perfusion with GH110B (post) for kidneys 1–3. Kidney 4 was a control that was perfused similarly without addition of enzyme and was the biological pair of kidney 3. Scale bar represents 10 μm. **b**,**c** Quantification of **b** B antigen and **c** H antigen staining in **a** shown as median integrated density values with 95 per cent confidence intervals for six randomly selected fields of view from each cortex biopsy. *P < 0.050, †*P* < 0.001 (Student's t-test).

## Discussion

The principle of antigen removal via NMP is applicable to all solid organs, and it could also be achieved during hypothermic machine perfusion if enzyme activity were optimized at lower temperatures^13^. Overall, machine perfusion offers an excellent strategy for treating an isolated organ before transplantation, removing the therapeutic challenges of specific targeting to the organ of interest, off-target effects, and dosage concerns, which arise when treating *in vivo*. Recent work used NMP in combination with FpGalNAc deacetylase and FpGalactosaminidase to remove up to 97 per cent of blood group A antigens from type A1 lungs^12^.

A potential limitation of the strategy described here is the transient removal of blood group antigens at the phenotypic but not genetic level. It is therefore expected that antigenic glycans will be renewed within a relatively short time frame and may present an immunogenic challenge to the recipient. However, the host immunological response to such a challenge may not be a significant clinical problem in view of the documented phenomenon of graft accommodation in ABOi transplantation. This describes the acquired resistance of an organ to antibody-mediated rejection following transplantation after recurrence of high antibody titres. The organs typically show normal histology and have glomerular filtration rates similar to those of ABOc kidneys^14^. It has been hypothesized that removal of incompatible blood group antigens at transplantation may prevent immediate hyperacute rejection and induce accommodation in the graft.

A further consideration to this work is the observed decrease in ABOi kidney transplants in favour of living kidney sharing schemes^15^. Such schemes allow ABOi donor–recipient pairs to be registered in a national scheme to allocate transplants between compatible pairs. Certain blood groups of recipients (mainly type O) and donors (mainly type AB) accumulate on waiting lists (lack of supply or demand respectively). In contrast, enzymatic blood group conversion provides a strategy to allow full use of the growing deceased donor organ supply to match the continuing demand for compatible organs^16^.

This study is limited by the small number of kidneys investigated, alongside inconsistent enzyme efficiency and the degree of variability in antigen removal. Inter-organ variability reflects the inherently heterogeneric nature of both surface blood group antigen expression and organ quality. Cold ischaemia time, donor past medical history, and donor age all contribute to the quality of perfusion and likely the efficacy of antigen removal. This research was the first step toward removing the ABO barrier from the field of kidney transplantation.

## Supplementary Material

znac293_Supplementary_Data
