# Supplementary material for: Enzymatic blood group conversion of human kidneys during ex vivo normothermic machine perfusion
Source: Br J Surg. 2022 Aug 30;110(2):133–7. doi: 10.1093/bjs/znac293 (PMC10364487; doi:10.1093/bjs/znac293)
Supplement: znac293_Supplementary_Data [file znac293_supplementary_data.docx]

**Supplementary Methods**

***Ethical approval***

Five human kidney cortical biopsies were obtained from kidneys determined to be unsuitable for transplantation and declined by UK transplant centres (3 x DBD, 2 x DCD). Four further human kidneys, including one pair, (3 x DBD, 1 x DCD) were recruited to this study for perfusion. Ethical approval for use of human tissue in these experiments was obtained from the National Ethics Committee (NRES: 15/NE/0408) and by NHSBT. Following retrieval, the kidneys were flushed with cold preservation solution and underwent static cold storage on ice until sample collection or perfusion. The kidneys used in this study were all of blood group B (8 x B positive, 1 x B negative).

***In vitro antigen removal in human kidney sections and immunofluorescence staining***

Formalin-fixed paraffin-embedded (FFPE) sections of human kidney cortex tissue 7μm thick were deparaffinised and rehydrated before a 1hr incubation at 37^o^C in varying concentrations of GH110B (250μg/ml, 25μg/ml, 2.5μg/ml, 0.25μg/ml, or 0μg/ml) diluted in 200μl acellular perfusate (see Supplementary Material for full composition). Sections were washed and then stained sequentially with primary antibodies then secondary antibodies diluted in PBS + 0.5% Tween20 each for 1hr at room temperature (full list included in Supplementary Table 1).

***Fluorescence microscopy, image analysis, and quantification***

All FFPE sections were imaged with a Leica SP5 confocal microscope. For each section, six random fields of view were imaged, and all image analysis was subsequently completed using FIJI software^23^. Briefly, for quantification of antigen staining, the integrated density of anti-B or *Ulex europaeus* fluorescence staining was recorded above a threshold value of background staining obtained from appropriately stained negative controls. Percentage antigen loss was calculated relative to untreated sections (for *in vitro* antigen removal), or biopsies taken before enzyme addition (for *ex vivo* antigen removal).

***Ex vivo normothermic machine perfusion***

Four human kidneys underwent NMP as previously described^24^ using an acellular perfusate (see Supplementary Material for composition). To 400ml of perfusate, 1mg of GH110B (CZ1050; NZYTech, Lisbon, Portugal) was added to three of the kidneys. One paired kidney (control) was perfused without the addition of the enzyme. Kidneys were perfused for 5hrs at 37^o^C with cortical wedge biopsies taken every hour.

***Histological staining and grading***

All FFPE tissue sections were stained with haematoxylin and eosin (H&E) by the Human Research Tissue Bank at Addenbrooke’s Hospital. Five random fields of view per tissue section were imaged with an Olympus IX81 inverted microscope (100x magnification). Histological signs of renal damage (tubular flattening, vacuolisation, tubular debris, epithelial flattening, glomerular shrinkage, and interstitial fibrosis) were graded based on the percentage of parenchyma affected: no acute kidney injury (Grade 0: <5%), mild injury (Grade 1: 6-25%), moderate injury (Grade 2: 26-50%), and severe injury (Grade 3: >50%).

***Statistical analysis***

For image analysis, a one-way repeated measure ANOVA with Geisser-Greenhouse correction for sphericity was completed. A post-hoc Tukey’s multiple comparisons test was used to compare between groups. A Student’s t-test was used to compare between timepoints. Statistical analysis was completed using GraphPad Prism version 9.0 (GraphPad Software, San Diego, California USA). Significance level was defined as p < 0.050.

|  | **Host** | **Reactivity** | **Isotype** | **Conjugate** | **Dilution** | **Catalogue no.** |
| --- | --- | --- | --- | --- | --- | --- |
| **Anti-B** | Mouse | Human | Monoclonal IgM | Unconjugated | 1:100 | Z011; Quotient, Utah, USA |
| **Anti-CD31** | Rabbit | Human | Polyclonal IgG | Unconjugated | 1:50 | ab28364; Abcam, Cambridge, UK |
| ***Ulex europaeus* I lectin** | N/A | N/A | N/A | Biotin | 1:50 | GTX01511; GeneTex, CA, USA |
| **Anti-mouse IgM-AF555** | Goat | Mouse | Polyclonal IgG | AF555 | 1:500 | A-21426; Invitrogen, CA, USA |
| **Anti-rabbit IgG-AF488** | Goat | Rabbit | Polyclonal IgG | AF488 | 1:1000 | A-11008; Invitrogen, CA, USA |
| **Streptavidin-AF647** | N/A | N/A | N/A | AF647 | 1:1000 | S32357; Invitrogen, CA, USA |
|  |  |  |  |  |  |  |

**List of antibodies and lectins**

**Acellular perfusate composition (total 400ml):**

| **Component** | **Amount** |
| --- | --- |
| Ringer’s solution | 285 ml |
| Human serum albumin (20%) | 100 ml |
| Sodium bicarbonate | Variable – to normalise pH |
| Mannitol | 5 ml |
| Calcium gluconate (10%) | 10 ml |

**Supplements from infusions:**

| **Component** | | **Amount** |
| --- | --- | --- |
| Prostacyclin | | 16 ml in 100 ml saline |
| Synthamin 17 with multivitamins: | | 10ml/hr |
|  | Insulin | 100 units |
|  | Sodium bicarbonate | 15 ml |
| Glucose | | 2ml/hr |

**Table S1.** Donor characteristics for perfused kidneys

|  | **Kidney 1** | **Kidney 2** | **Kidney Pair**  **(3 and 4)** |
| --- | --- | --- | --- |
| **Age (years)** | 79 | 75 | 65 |
| **Sex** | Not recorded | Female | Male |
| **Blood group** | B positive | B positive | B positive |
| **Left/Right** | Left | Right | Left (3); Right (4) |
| **DCD/DBD** | DCD | DBD | DBD |
| **WIT (mins)** | 10 | 0 | 0 |
| **CIT (mins)** | 654 | 879 | 1691 |
| **Cause of death** | Cardiac arrest | Intracranial haemorrhage | Myocardial infarction |
| **Reason for discard** | Renal cell carcinoma | Prolonged CIT | Nodule on lung |
| **Past medical history** | Pulmonary disease | Hypertension, cancer, liver disease | Diabetes |

DCD – donation after circulatory death; DBD – donation after brain death; WIT – warm ischaemic time; CIT – cold ischaemic time.

**Table S2.** Haemodynamic parameters during perfusion

|  | **Kidney 1** | **Kidney 2** | | **Kidney 3** | **Kidney 4** | | |
| --- | --- | --- | --- | --- | --- | --- | --- |
| **Length of perfusion with enzyme (mins)** | 300 | 300 | | 300 | 300 | | |
| **MAP in (mmHg)** | 52.6 ± 0.8 | 52.2 ± 1.0 | | 55.2 ± 11.0 | 67.7 ± 6.0 | | |
| **RBF (ml/100g/min)** | 312.8 ± 19.1 | 502.9 ± 11.4 | | 423.5 ± 46.7 | 248.8 ± 112.8 | | |
| **IRR (mmHg/ml/min/100g)** | 0.169 ± 0.014 | 0.104 ± 0.003 | | 0.134 ± 0.045 | 0.361 ± 0.244 | | |
| **Temperature (^o^C)** | 36.4 ± 0.1 | 36.4 ± 0.3 | | 37.4 ± 0.0 | 37.4 ± 0.0 | | |
| **Total urine output (ml)** | 3 | 70 | | 28 | 5 | | |
| **Arterial pH** | 7.50 ± 0.03 | 7.49 ± 0.17 | | 7.38 ± 0.03 | 7.46 ± 0.41 | | |
| **Arterial pO2 (kPa)** | 72.2 ± 8.8 | 76.4 ± 2.6 | | 80.9 ± 1.1 | 79.7 ± 1.9 | | |
| **Venous pO2 (kPa)** | 39.9^a^ | 41.9 ± 3.1 | | 54.0 ± 1.8 | 44.3 ± 7.7 | | |
|  |  | |  | | |  |  |

Data are presented as mean ± standard deviation.

^a^ Only one value for venous pO_2_ was obtained

MAP – mean arterial pressure; RBF – renal blood flow; IRR – intrarenal resistance.

**Figure S1.** Study design. (A) *In vitro* antigen removal using the α-galactosidase GH110B from *Bacteroides fragilis* in five human blood group B kidney cortex sections. Antigen removal was quantified using immunofluorescence staining of B and H antigens. (B) Blood group conversion of three blood group B human kidneys during *ex vivo* normothermic machine perfusion. Kidneys were perfused with an acellular solution supplemented with 2.5μg/ml GH110B at 37^o^C for 5hrs. Cortical biopsies were taken each hour for quantification of B antigen removal and H antigen emergence.

FFPE – Formalin-fixed paraffin-embedded. NMP – normothermic machine perfusion.

**
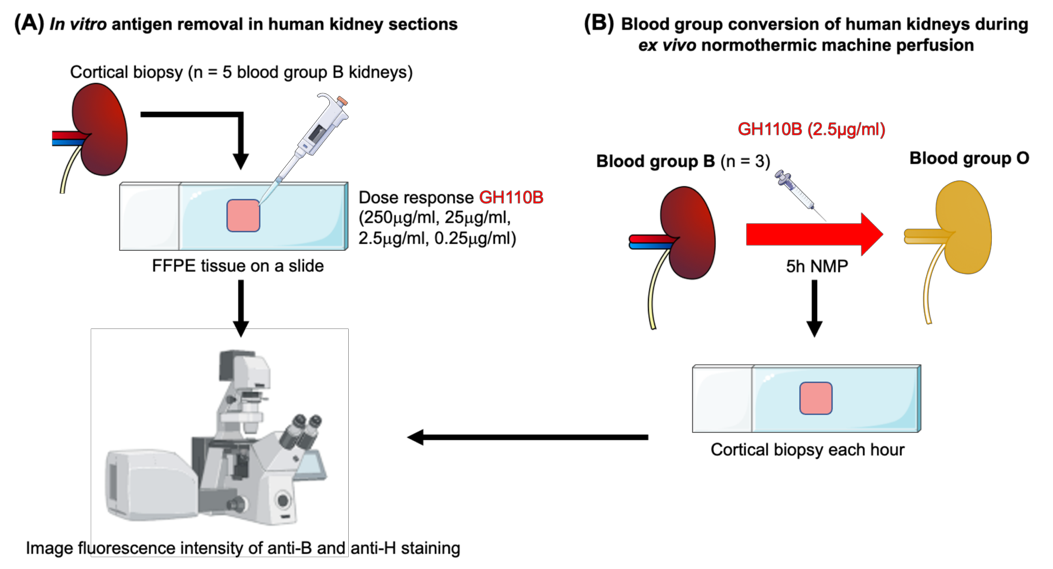
**

**Figure S2**. A schematic representing blood group A and B antigen biosynthesis from the core H antigen oligosaccharide by the GTA or GTB enzymes, respectively. “A”-zymes and “B”-zymes (the latter including the α-galactosidase GH110B from *B. fragilis*) can be used to convert A and B antigens back to the H antigen by removing the terminal N-acetylgalactosamine (GalNAc) or galactose moiety, respectively.

Gal – Galactose; GlcNAc – N-acetylglucosamine; Fuc – fucose; GalNAc – N-acetylgalactosamine; GTA – α-1-3-N-acetylgalactosaminyltransferase; GTB - α-1-3-galactosyltransferase.

**
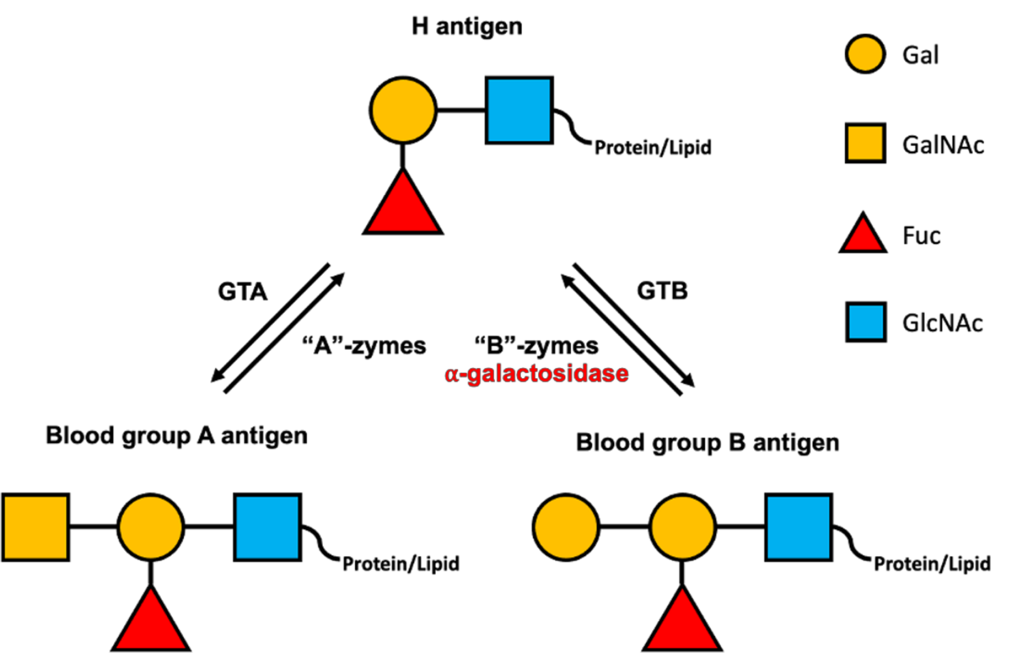
**

**Figure S3.** Perfusion parameters during NMP. (A) Renal blood flow (RBF), and (B) intrarenal resistance (IRR) during 5hrs acellular NMP for all four perfused kidneys (3x treated - left, 1 x untreated control – right).

RBF – Renal Blood Flow; IRR – Intrarenal Resistance; NMP – normothermic machine perfusion.

**
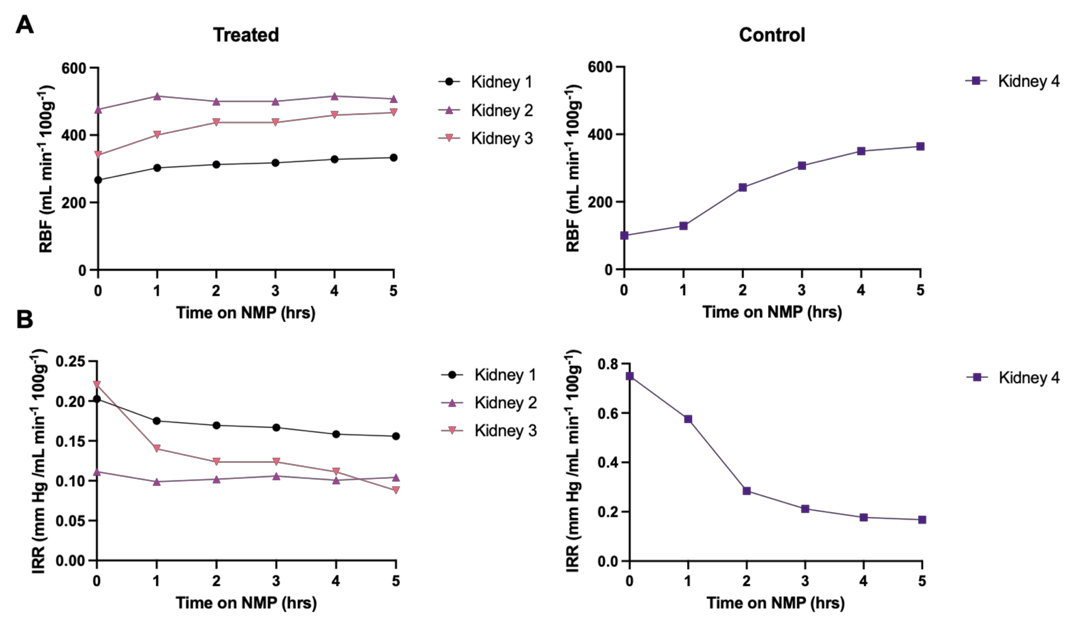
**

**Figure S4.** Histological assessment of perfused kidneys. (A) Summary of histological grading for kidney injury during perfusion for the four perfused kidneys (3 x treated, 1 x control). (B) Representative histology images stained with haematoxylin and eosin of kidney 3 and kidney 4 (a biological pair) at 0hr (pre-enzyme addition) and 5hrs after perfusion with GH110B (treated) or no enzyme (control). Black arrows indicate interstitial fibrosis; blue arrows represent tubular vacuolation, and green arrows indicate epithelial shedding and tubular debris. Scale bar represents 200μm.

AKI – acute kidney injury.

**
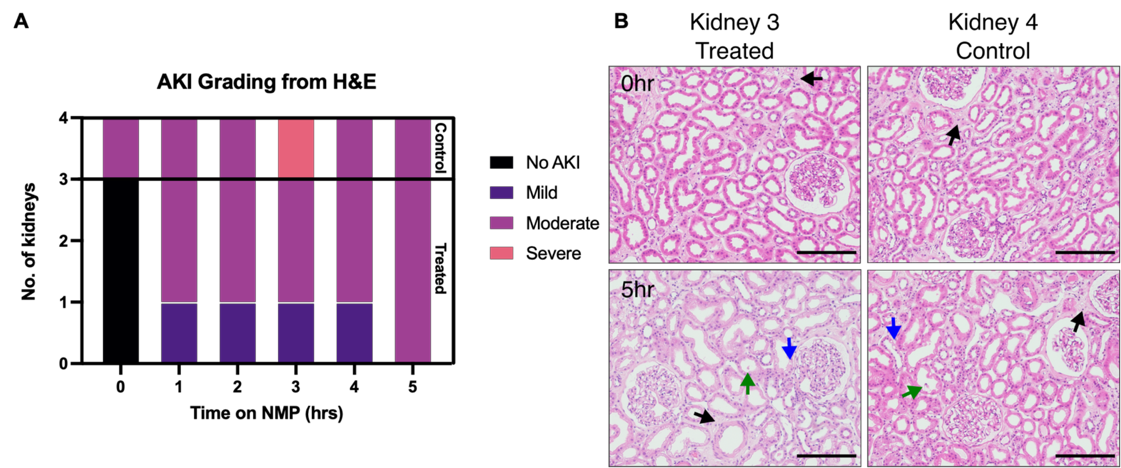
**

**Figure S5.** Enzymatic B antigen removal in Kidney 3 during *ex vivo* NMP at all timepoints. Immunofluorescence images showing staining of blood group B antigens (purple) and H antigens (green) before enzyme addition (0hr), and at hourly intervals during NMP with GH110B. A composite of B and H antigen staining is shown in the right-hand panels. Scale bar indicates 100μm.

**
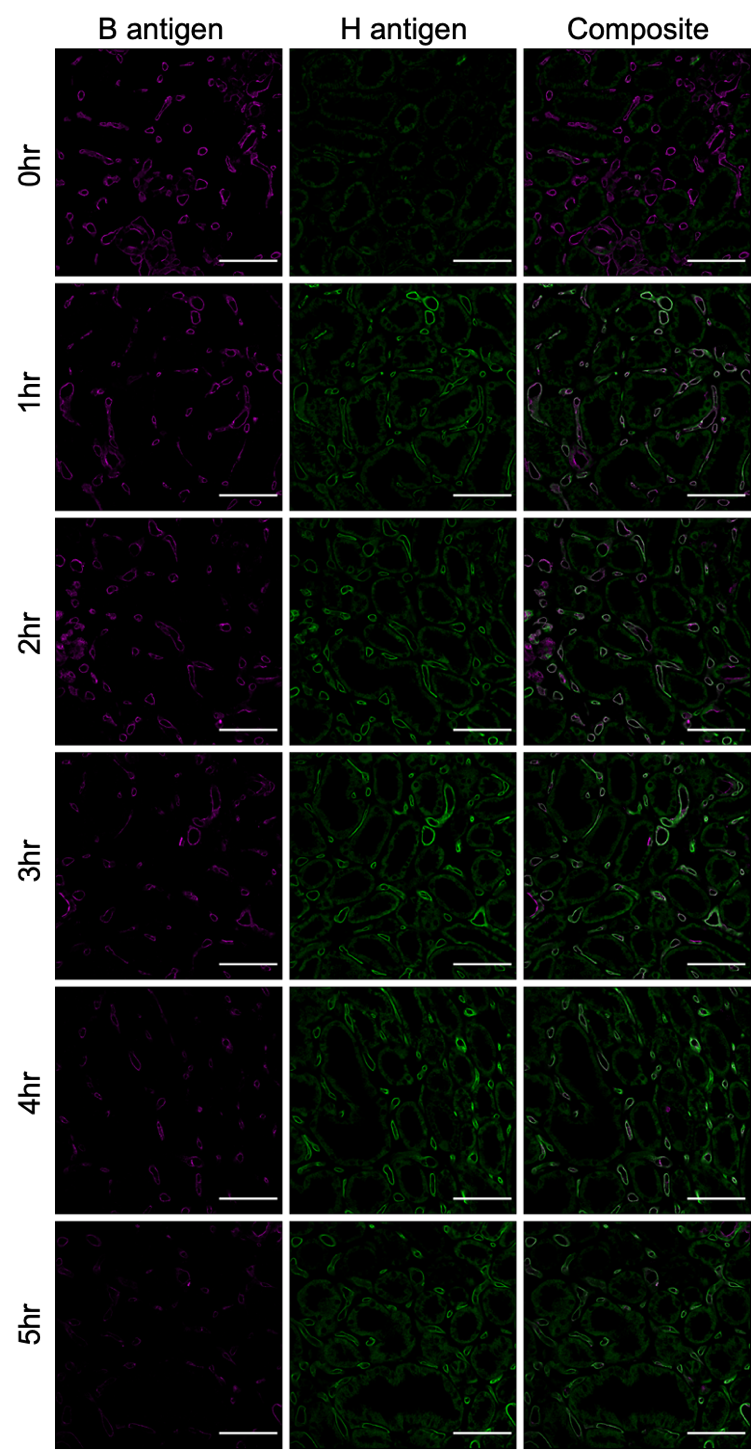
**
